# Level of lipopolysaccharide-binding protein and microbial landscape with account of severity of sepsis syndromes in polytrauma

**DOI:** 10.1186/cc12229

**Published:** 2013-03-19

**Authors:** IM Ustyantseva, OI Khokhlova, OV Petukhova, YA Zhevlakova

**Affiliations:** 1 Federal Scientific Clinical Center of Miners' Health Protection, Leninsk-Kuznetsky, Russia

## Introduction

The aim was estimation of the clinical and predictive significance of the level of lipopolysaccharide-binding protein (LBP) in blood serum and examination of microbial landscape with account of severity of sepsis syndromes in polytrauma.

## Methods

Clinical examination was performed, which included 99 patients with polytrauma according to sepsis syndrome: SIRS (*n = *18), local infection (*n = *36), sepsis (*n = *27), severe sepsis (*n = *12), septic shock (*n = *6) to the criteria of ACCP/SCCM. The microorganism identification was performed using iEMS Reader MF (Labsystems) with the La Chema multimicrotests. The content of LBP in blood serum was assessed with IMMULITE ONE (USA) with the reagents DPC (USA). Statistical analysis of the data was performed with Statistica 6.0. The numerical characteristics of variables are presented as Me (LQ to UQ). The analysis of differences was carried with the Kruskal-Wallis test for multiple comparison of independent groups, with Friedman one-way analysis of variance. The differences were statistically significant with *P <*0.05.

## Results

In 81% of the critically ill patients with polytrauma the post-traumatic period was accompanied with development of infectious complications, Gram-negative (*K. pneumoniae*, Acinetobacter spp., *E. coli*) and Gram-positive (*S. Epidermidis, S. aureus*). Sepsis was diagnosed on 8 to 10 days in 45% of the patients. The significant increase of LPS-BP was found in the first 3 days of the follow-up, compared with the control values (6.7 times higher in SIRS group (χ^2^(*n = *18, df = 3) = 52.8666, *P <*0.001); 9.9 times higher in the group with local infection (χ^2^(*n = *36, df = 3) = 91.6629, *P <*0.001); 15.2 times higher in the sepsis group; 20.5 times higher in the severe sepsis group; 47.3 times higher in the septic shock group (χ^2^(*n = *6, df = 3) = 11.0339, *P *= 0.0115)), whereas the first positive results of the microbiological examination were obtained only on 5 to 7 days. The diagnostic sensitivity of threshold concentration of LBP in blood serum (335 mkg/ml) was 84%, diagnostic specificity was 88% (ROC curve: 0.88).

## Conclusion

The high incidence of the diagnostic levels of LBP in blood serum in patients with sepsis in the early term, before microbiological confirmation of infection, allows one to use this parameter as an early marker of development of purulent septic complications conditioned by Gram-negative microflora.

